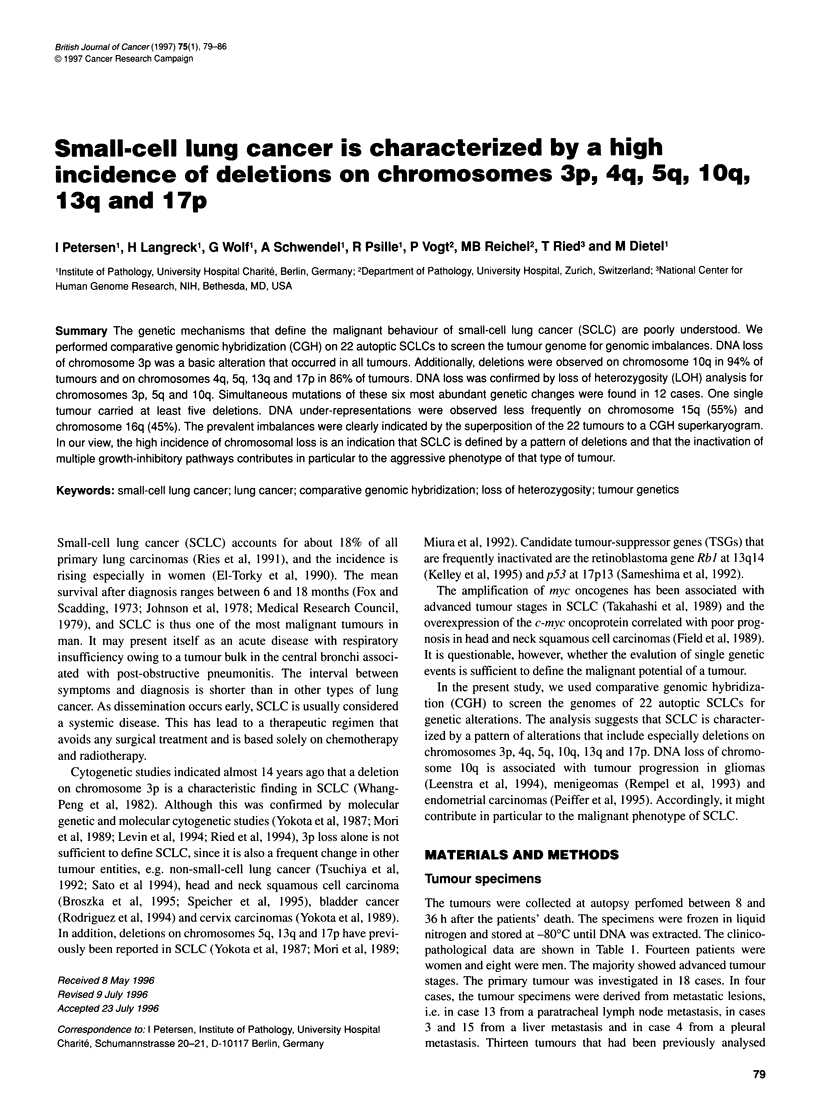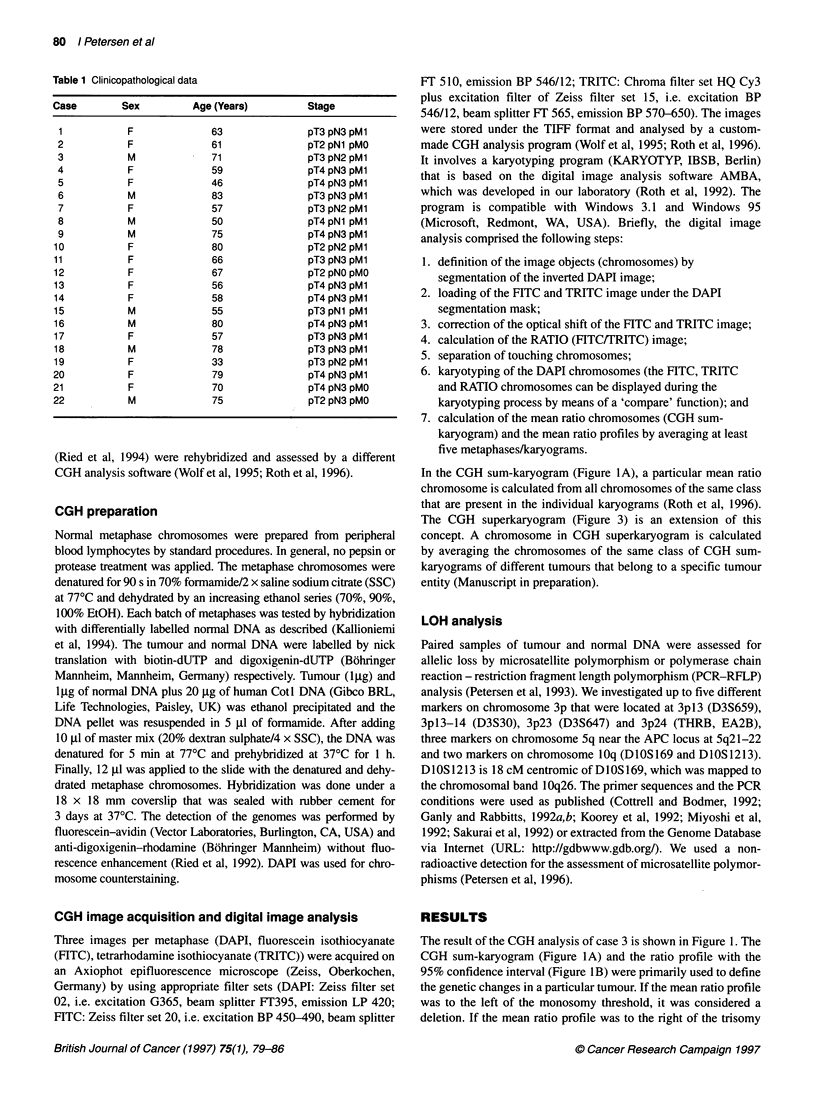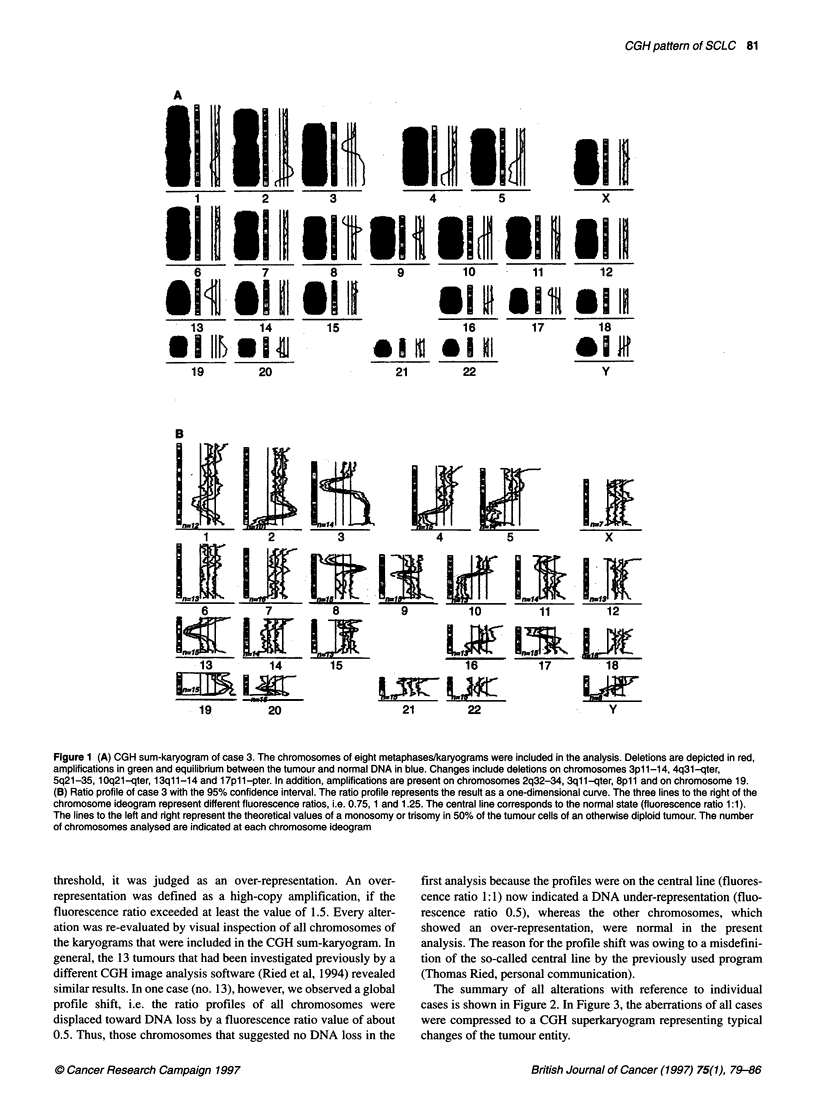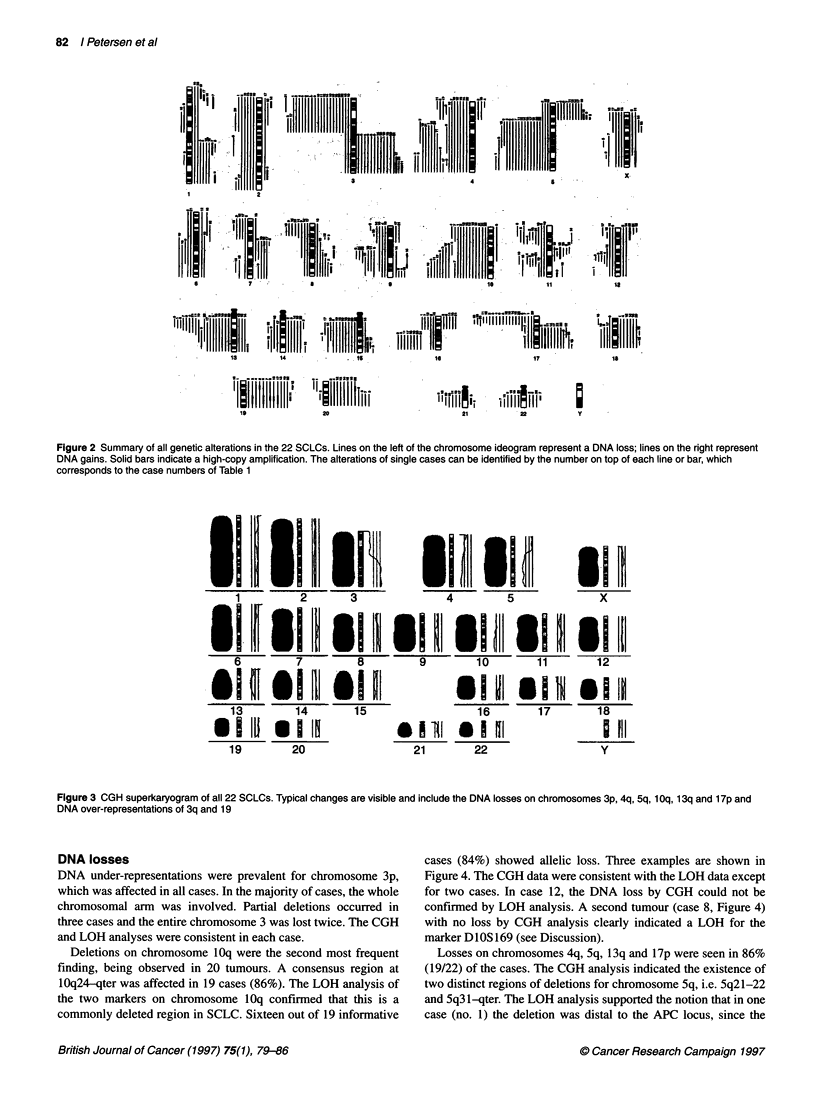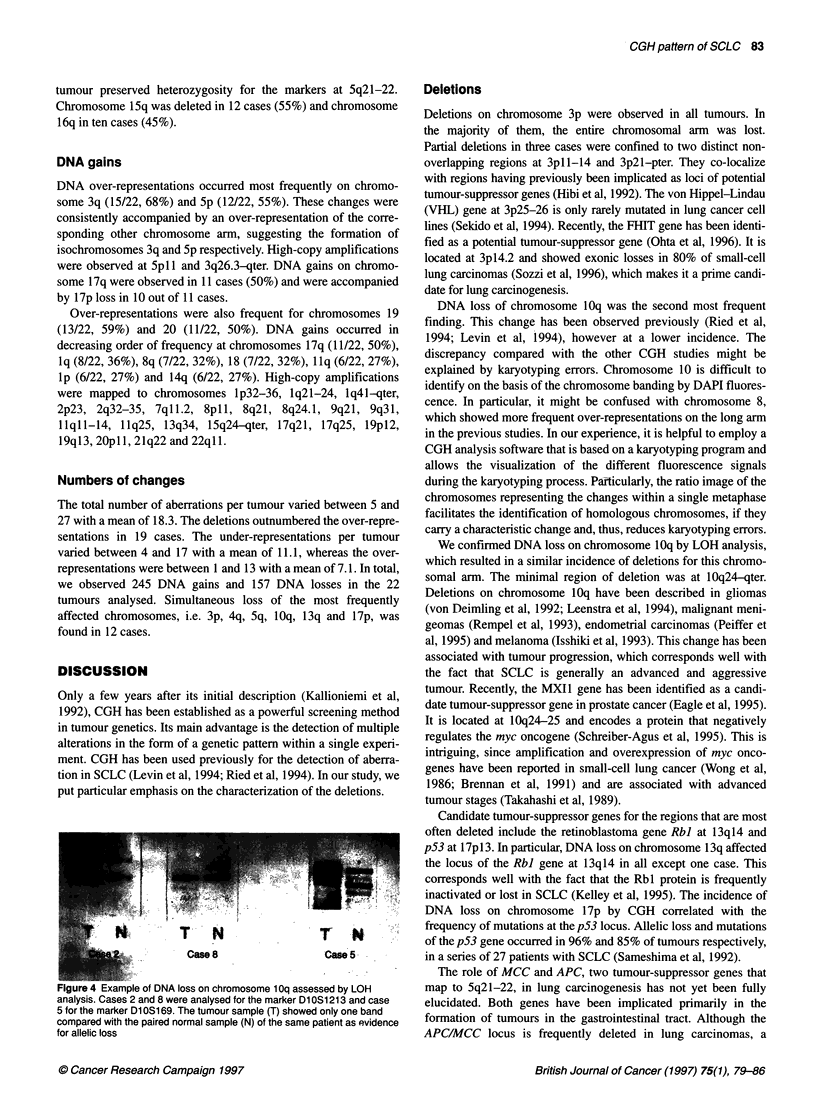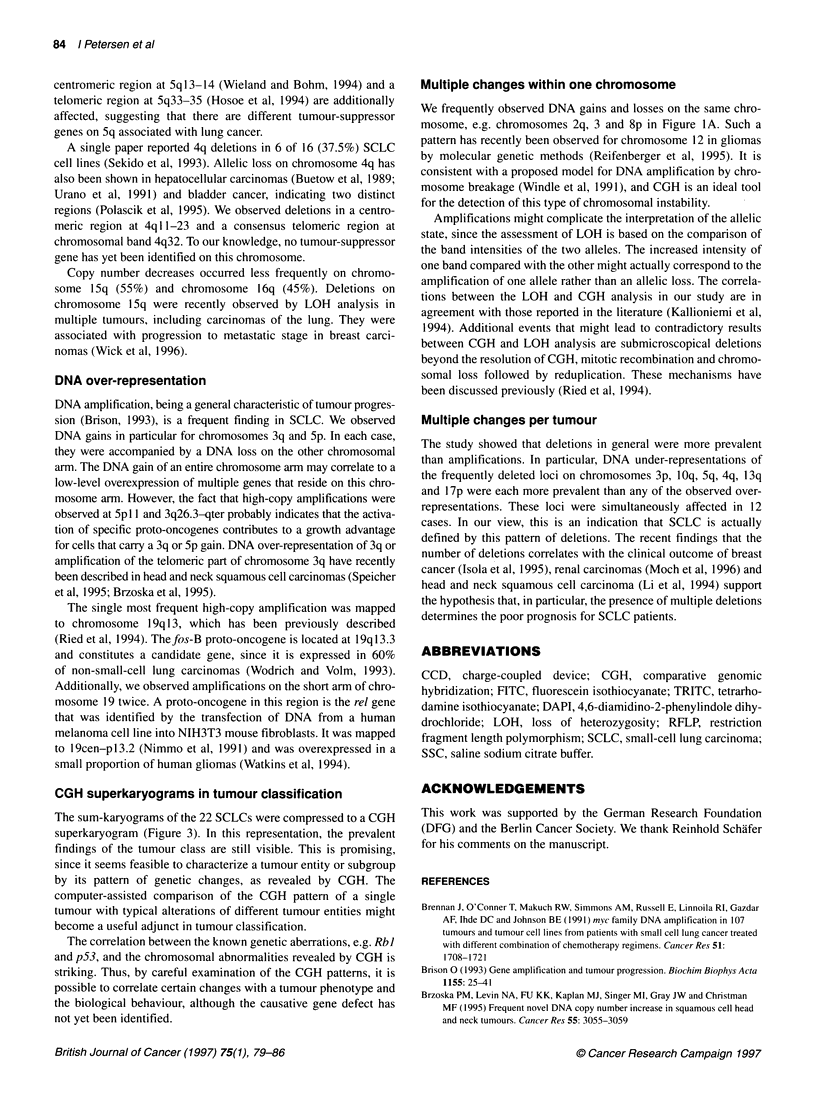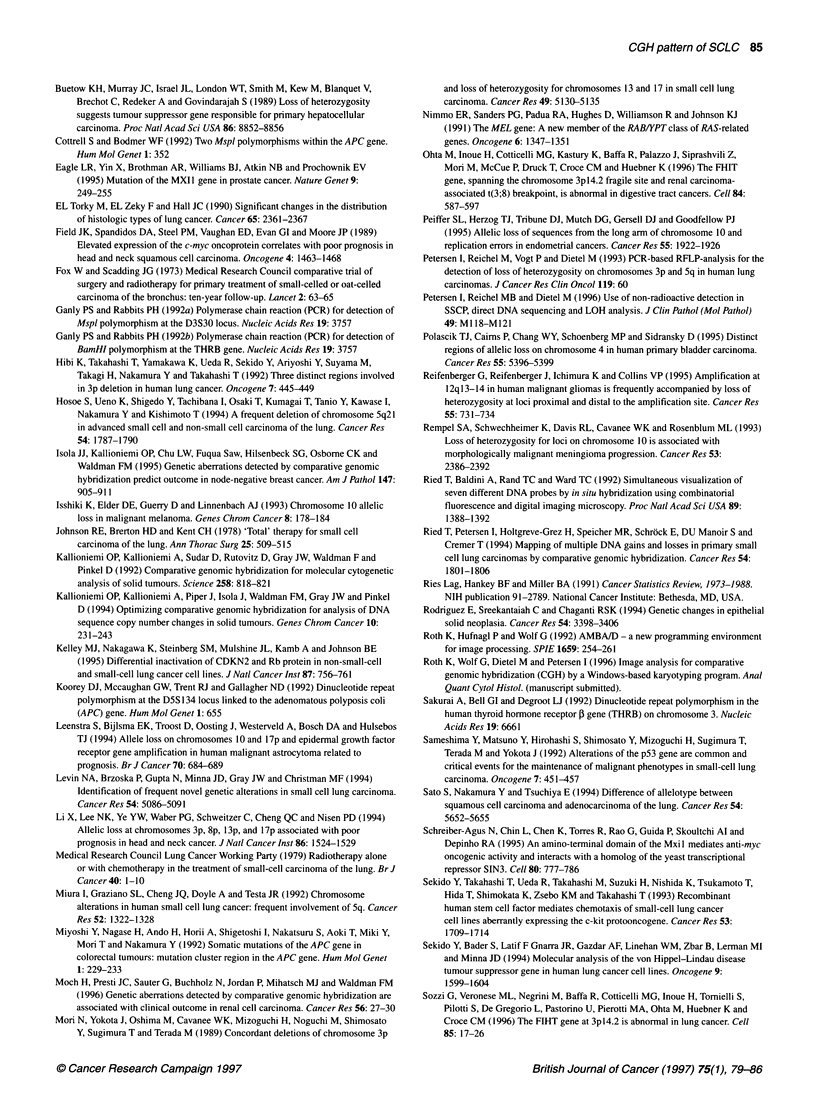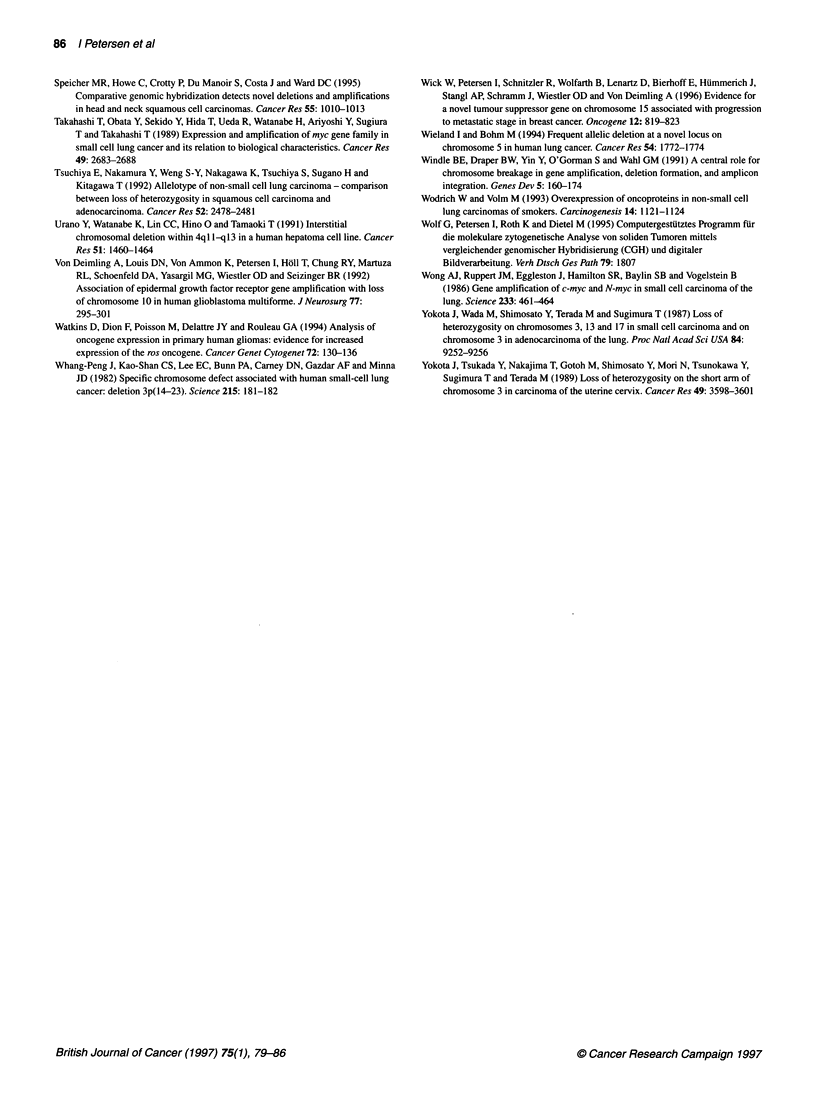# Small-cell lung cancer is characterized by a high incidence of deletions on chromosomes 3p, 4q, 5q, 10q, 13q and 17p.

**DOI:** 10.1038/bjc.1997.13

**Published:** 1997

**Authors:** I. Petersen, H. Langreck, G. Wolf, A. Schwendel, R. Psille, P. Vogt, M. B. Reichel, T. Ried, M. Dietel

**Affiliations:** Institute of Pathology, University Hospital CharitÃ©, Berlin, Germany.

## Abstract

**Images:**


British Journal of Cancer (1997) 75(1), 79–86
© 1997 Cancer Research Campaign

### Small-cell lung cancer is characterized by a high incidence of deletions on chromosomes 3p, 4q, 5q, 10q, 13q and 17p

**I Petersen[1], H Langreck[1], G Wolf[1], A Schwendel[1], R Psille[1], P Vogt[2], MB Reichel[2], T Ried[3] and M Dietel[1]**

[1]Institute of Pathology, University Hospital Charité, Berlin, Germany; [2]Department of Pathology, University Hospital, Zurich, Switzerland; [3]National Center for Human Genome Research, NIH, Bethesda, MD, USA

**Summary** The genetic mechanisms that define the malignant behaviour of small-cell lung cancer (SCLC) are poorly understood. We performed comparative genomic hybridization (CGH) on 22 autoptic SCLCs to screen the tumour genome for genomic imbalances. DNA loss of chromosome 3p was a basic alteration that occurred in all tumours. Additionally, deletions were observed on chromosome 10q in 94% of tumours and on chromosomes 4q, 5q, 13q and 17p in 86% of tumours. DNA loss was confirmed by loss of heterozygosity (LOH) analysis for chromosomes 3p, 5q and 10q. Simultaneous mutations of these six most abundant genetic changes were found in 12 cases. One single tumour carried at least five deletions. DNA under-representations were observed less frequently on chromosome 15q (55%) and chromosome 16q (45%). The prevalent imbalances were clearly indicated by the superposition of the 22 tumours to a CGH superkaryogram. In our view, the high incidence of chromosomal loss is an indication that SCLC is defined by a pattern of deletions and that the inactivation of multiple growth-inhibitory pathways contributes in particular to the aggressive phenotype of that type of tumour.

**Keywords:** small-cell lung cancer; lung cancer; comparative genomic hybridization; loss of heterozygosity; tumour genetics

Small-cell lung cancer (SCLC) accounts for about 18% of all primary lung carcinomas (Ries et al, 1991), and the incidence is rising especially in women (El-Torky et al, 1990). The mean survival after diagnosis ranges between 6 and 18 months (Fox and Scadding, 1973; Johnson et al, 1978; Medical Research Council, 1979), and SCLC is thus one of the most malignant tumours in man. It may present itself as an acute disease with respiratory insufficiency owing to a tumour bulk in the central bronchi associated with post-obstructive pneumonitis. The interval between symptoms and diagnosis is shorter than in other types of lung cancer. As dissemination occurs early, SCLC is usually considered a systemic disease. This has lead to a therapeutic regimen that avoids any surgical treatment and is based solely on chemotherapy and radiotherapy.

Cytogenetic studies indicated almost 14 years ago that a deletion on chromosome 3p is a characteristic finding in SCLC (Whang-Peng et al, 1982). Although this was confirmed by molecular genetic and molecular cytogenetic studies (Yokota et al, 1987; Mori et al, 1989; Levin et al, 1994; Ried et al, 1994), 3p loss alone is not sufficient to define SCLC, since it is also a frequent change in other tumour entities, e.g. non-small-cell lung cancer (Tsuchiya et al, 1992; Sato et al 1994), head and neck squamous cell carcinoma (Broszka et al, 1995; Speicher et al, 1995), bladder cancer (Rodriguez et al, 1994) and cervix carcinomas (Yokota et al, 1989). In addition, deletions on chromosomes 5q, 13q and 17p have previously been reported in SCLC (Yokota et al, 1987; Mori et al, 1989; Miura et al, 1992). Candidate tumour-suppressor genes (TSGs) that are frequently inactivated are the retinoblastoma gene *Rb1* at 13q14 (Kelley et al, 1995) and *p53* at 17p13 (Sameshima et al, 1992).

*Received 8 May 1996*
*Revised 9 July 1996*
*Accepted 23 July 1996*

*Correspondence to:* I Petersen, Institute of Pathology, University Hospital Charité, Schumannstrasse 20–21, D-10117 Berlin, Germany

The amplification of *myc* oncogenes has been associated with advanced tumour stages in SCLC (Takahashi et al, 1989) and the overexpression of the *c-myc* oncoprotein correlated with poor prognosis in head and neck squamous cell carcinomas (Field et al, 1989). It is questionable, however, whether the evalution of single genetic events is sufficient to define the malignant potential of a tumour.

In the present study, we used comparative genomic hybridization (CGH) to screen the genomes of 22 autoptic SCLCs for genetic alterations. The analysis suggests that SCLC is characterized by a pattern of alterations that include especially deletions on chromosomes 3p, 4q, 5q, 10q, 13q and 17p. DNA loss of chromosome 10q is associated with tumour progression in gliomas (Leenstra et al, 1994), menigeomas (Rempel et al, 1993) and endometrial carcinomas (Peiffer et al, 1995). Accordingly, it might contribute in particular to the malignant phenotype of SCLC.

### MATERIALS AND METHODS

### Tumour specimens

The tumours were collected at autopsy perfomed between 8 and 36 h after the patients' death. The specimens were frozen in liquid nitrogen and stored at –80°C until DNA was extracted. The clinicopathological data are shown in Table 1. Fourteen patients were women and eight were men. The majority showed advanced tumour stages. The primary tumour was investigated in 18 cases. In four cases, the tumour specimens were derived from metastatic lesions, i.e. in case 13 from a paratracheal lymph node metastasis, in cases 3 and 15 from a liver metastasis and in case 4 from a pleural metastasis. Thirteen tumours that had been previously analysed

Table 1 Clinicopathological data

| Case | Sex | Age (Years) | Stage |
|---|---|---|---|
| 1 | F | 63 | pT3 pN3 pM1 |
| 2 | F | 61 | pT2 pN1 pM0 |
| 3 | M | 71 | pT3 pN2 pM1 |
| 4 | F | 59 | pT4 pN3 pM1 |
| 5 | F | 46 | pT4 pN3 pM1 |
| 6 | M | 83 | pT3 pN3 pM1 |
| 7 | F | 57 | pT3 pN2 pM1 |
| 8 | M | 50 | pT4 pN1 pM1 |
| 9 | M | 75 | pT4 pN3 pM1 |
| 10 | F | 80 | pT2 pN2 pM1 |
| 11 | F | 66 | pT3 pN3 pM1 |
| 12 | F | 67 | pT2 pN0 pM0 |
| 13 | F | 56 | pT4 pN3 pM1 |
| 14 | F | 58 | pT4 pN3 pM1 |
| 15 | M | 55 | pT3 pN1 pM1 |
| 16 | M | 80 | pT4 pN3 pM1 |
| 17 | F | 57 | pT3 pN3 pM1 |
| 18 | M | 78 | pT3 pN3 pM1 |
| 19 | F | 33 | pT3 pN2 pM1 |
| 20 | F | 79 | pT4 pN3 pM1 |
| 21 | F | 70 | pT4 pN3 pM0 |
| 22 | M | 75 | pT2 pN3 pM0 |

(Ried et al, 1994) were rehybridized and assessed by a different CGH analysis software (Wolf et al, 1995; Roth et al, 1996).

### CGH preparation

Normal metaphase chromosomes were prepared from peripheral blood lymphocytes by standard procedures. In general, no pepsin or protease treatment was applied. The metaphase chromosomes were denatured for 90 s in 70% formamide/2 × saline sodium citrate (SSC) at 77°C and dehydrated by an increasing ethanol series (70%, 90%, 100% EtOH). Each batch of metaphases was tested by hybridization with differentially labelled normal DNA as described (Kallioniemi et al, 1994). The tumour and normal DNA were labelled by nick translation with biotin-dUTP and digoxigenin-dUTP (Böhringer Mannheim, Mannheim, Germany) respectively. Tumour (1µg) and 1µg of normal DNA plus 20 µg of human Cot1 DNA (Gibco BRL, Life Technologies, Paisley, UK) was ethanol precipitated and the DNA pellet was resuspended in 5 µl of formamide. After adding 10 µl of master mix (20% dextran sulphate/4 × SSC), the DNA was denatured for 5 min at 77°C and prehybridized at 37°C for 1 h. Finally, 12 µl was applied to the slide with the denatured and dehydrated metaphase chromosomes. Hybridization was done under a 18 × 18 mm coverslip that was sealed with rubber cement for 3 days at 37°C. The detection of the genomes was performed by fluorescein–avidin (Vector Laboratories, Burlington, CA, USA) and anti-digoxigenin–rhodamine (Böhringer Mannheim) without fluorescence enhancement (Ried et al, 1992). DAPI was used for chromosome counterstaining.

### CGH image acquisition and digital image analysis

Three images per metaphase (DAPI, fluorescein isothiocyanate (FITC), tetrarhodamine isothiocyanate (TRITC)) were acquired on an Axiophot epifluorescence microscope (Zeiss, Oberkochen, Germany) by using appropriate filter sets (DAPI: Zeiss filter set 02, i.e. excitation G365, beam splitter FT395, emission LP 420; FITC: Zeiss filter set 20, i.e. excitation BP 450–490, beam splitter FT 510, emission BP 546/12; TRITC: Chroma filter set HQ Cy3 plus excitation filter of Zeiss filter set 15, i.e. excitation BP 546/12, beam splitter FT 565, emission BP 570–650). The images were stored under the TIFF format and analysed by a custom-made CGH analysis program (Wolf et al, 1995; Roth et al, 1996). It involves a karyotyping program (KARYOTYP, IBSB, Berlin) that is based on the digital image analysis software AMBA, which was developed in our laboratory (Roth et al, 1992). The program is compatible with Windows 3.1 and Windows 95 (Microsoft, Redmont, WA, USA). Briefly, the digital image analysis comprised the following steps:

1. definition of the image objects (chromosomes) by segmentation of the inverted DAPI image;
2. loading of the FITC and TRITC image under the DAPI segmentation mask;
3. correction of the optical shift of the FITC and TRITC image;
4. calculation of the RATIO (FITC/TRITC) image;
5. separation of touching chromosomes;
6. karyotyping of the DAPI chromosomes (the FITC, TRITC and RATIO chromosomes can be displayed during the karyotyping process by means of a 'compare' function); and
7. calculation of the mean ratio chromosomes (CGH sum-karyogram) and the mean ratio profiles by averaging at least five metaphases/karyograms.

In the CGH sum-karyogram (Figure 1A), a particular mean ratio chromosome is calculated from all chromosomes of the same class that are present in the individual karyograms (Roth et al, 1996). The CGH superkaryogram (Figure 3) is an extension of this concept. A chromosome in CGH superkaryogram is calculated by averaging the chromosomes of the same class of CGH sum-karyograms of different tumours that belong to a specific tumour entity (Manuscript in preparation).

### LOH analysis

Paired samples of tumour and normal DNA were assessed for allelic loss by microsatellite polymorphism or polymerase chain reaction – restriction fragment length polymorphism (PCR–RFLP) analysis (Petersen et al, 1993). We investigated up to five different markers on chromosome 3p that were located at 3p13 (D3S659), 3p13–14 (D3S30), 3p23 (D3S647) and 3p24 (THRB, EA2B), three markers on chromosome 5q near the APC locus at 5q21–22 and two markers on chromosome 10q (D10S169 and D10S1213). D10S1213 is 18 cM centromic of D10S169, which was mapped to the chromosomal band 10q26. The primer sequences and the PCR conditions were used as published (Cottrell and Bodmer, 1992; Ganly and Rabbitts, 1992*a,b*; Koorey et al, 1992; Miyoshi et al, 1992; Sakurai et al, 1992) or extracted from the Genome Database via Internet (URL: http://gdbwww.gdb.org/). We used a non-radioactive detection for the assessment of microsatellite polymorphisms (Petersen et al, 1996).

### RESULTS

The result of the CGH analysis of case 3 is shown in Figure 1. The CGH sum-karyogram (Figure 1A) and the ratio profile with the 95% confidence interval (Figure 1B) were primarily used to define the genetic changes in a particular tumour. If the mean ratio profile was to the left of the monosomy threshold, it was considered a deletion. If the mean ratio profile was to the right of the trisomy

© Cancer Research Campaign 1997

**Figure 1** (**A**) CGH sum-karyogram of case 3. The chromosomes of eight metaphases/karyograms were included in the analysis. Deletions are depicted in red, amplifications in green and equilibrium between the tumour and normal DNA in blue. Changes include deletions on chromosomes 3p11–14, 4q31–qter, 5q21–35, 10q21–qter, 13q11–14 and 17p11–pter. In addition, amplifications are present on chromosomes 2q32–34, 3q11–qter, 8p11 and on chromosome 19. (**B**) Ratio profile of case 3 with the 95% confidence interval. The ratio profile represents the result as a one-dimensional curve. The three lines to the right of the chromosome ideogram represent different fluorescence ratios, i.e. 0.75, 1 and 1.25. The central line corresponds to the normal state (fluorescence ratio 1:1). The lines to the left and right represent the theoretical values of a monosomy or trisomy in 50% of the tumour cells of an otherwise diploid tumour. The number of chromosomes analysed are indicated at each chromosome ideogram threshold, it was judged as an over-representation. An over-representation was defined as a high-copy amplification, if the fluorescence ratio exceeded at least the value of 1.5. Every alteration was re-evaluated by visual inspection of all chromosomes of the karyograms that were included in the CGH sum-karyogram. In general, the 13 tumours that had been investigated previously by a different CGH image analysis software (Ried et al, 1994) revealed similar results. In one case (no. 13), however, we observed a global profile shift, i.e. the ratio profiles of all chromosomes were displaced toward DNA loss by a fluorescence ratio value of about 0.5. Thus, those chromosomes that suggested no DNA loss in the first analysis because the profiles were on the central line (fluorescence ratio 1:1) now indicated a DNA under-representation (fluorescence ratio 0.5), whereas the other chromosomes, which showed an over-representation, were normal in the present analysis. The reason for the profile shift was owing to a misdefinition of the so-called central line by the previously used program (Thomas Ried, personal communication).

The summary of all alterations with reference to individual cases is shown in Figure 2. In Figure 3, the aberrations of all cases were compressed to a CGH superkaryogram representing typical changes of the tumour entity.

© Cancer Research Campaign 1997 

Figure 2 Summary of all genetic alterations in the 22 SCLCs. Lines on the left of the chromosome ideogram represent a DNA loss; lines on the right represent DNA gains. Solid bars indicate a high-copy amplification. The alterations of single cases can be identified by the number on top of each line or bar, which corresponds to the case numbers of Table 1

Figure 3 CGH superkaryogram of all 22 SCLCs. Typical changes are visible and include the DNA losses on chromosomes 3p, 4q, 5q, 10q, 13q and 17p and DNA over-representations of 3q and 19

### DNA losses

DNA under-representations were prevalent for chromosome 3p, which was affected in all cases. In the majority of cases, the whole chromosomal arm was involved. Partial deletions occurred in three cases and the entire chromosome 3 was lost twice. The CGH and LOH analyses were consistent in each case.

Deletions on chromosome 10q were the second most frequent finding, being observed in 20 tumours. A consensus region at 10q24–qter was affected in 19 cases (86%). The LOH analysis of the two markers on chromosome 10q confirmed that this is a commonly deleted region in SCLC. Sixteen out of 19 informative cases (84%) showed allelic loss. Three examples are shown in Figure 4. The CGH data were consistent with the LOH data except for two cases. In case 12, the DNA loss by CGH could not be confirmed by LOH analysis. A second tumour (case 8, Figure 4) with no loss by CGH analysis clearly indicated a LOH for the marker D10S169 (see Discussion).

Losses on chromosomes 4q, 5q, 13q and 17p were seen in 86% (19/22) of the cases. The CGH analysis indicated the existence of two distinct regions of deletions for chromosome 5q, i.e. 5q21–22 and 5q31–qter. The LOH analysis supported the notion that in one case (no. 1) the deletion was distal to the APC locus, since the

© Cancer Research Campaign 1997

tumour preserved heterozygosity for the markers at 5q21–22. Chromosome 15q was deleted in 12 cases (55%) and chromosome 16q in ten cases (45%).

### DNA gains

DNA over-representations occurred most frequently on chromosome 3q (15/22, 68%) and 5p (12/22, 55%). These changes were consistently accompanied by an over-representation of the corresponding other chromosome arm, suggesting the formation of isochromosomes 3q and 5p respectively. High-copy amplifications were observed at 5p11 and 3q26.3–qter. DNA gains on chromosome 17q were observed in 11 cases (50%) and were accompanied by 17p loss in 10 out of 11 cases.

Over-representations were also frequent for chromosomes 19 (13/22, 59%) and 20 (11/22, 50%). DNA gains occurred in decreasing order of frequency at chromosomes 17q (11/22, 50%), 1q (8/22, 36%), 8q (7/22, 32%), 18 (7/22, 32%), 11q (6/22, 27%), 1p (6/22, 27%) and 14q (6/22, 27%). High-copy amplifications were mapped to chromosomes 1p32–36, 1q21–24, 1q41–qter, 2p23, 2q32–35, 7q11.2, 8p11, 8q21, 8q24.1, 9q21, 9q31, 11q11–14, 11q25, 13q34, 15q24–qter, 17q21, 17q25, 19p12, 19q13, 20p11, 21q22 and 22q11.

### Numbers of changes

The total number of aberrations per tumour varied between 5 and 27 with a mean of 18.3. The deletions outnumbered the over-representations in 19 cases. The under-representations per tumour varied between 4 and 17 with a mean of 11.1, whereas the over-representations were between 1 and 13 with a mean of 7.1. In total, we observed 245 DNA gains and 157 DNA losses in the 22 tumours analysed. Simultaneous loss of the most frequently affected chromosomes, i.e. 3p, 4q, 5q, 10q, 13q and 17p, was found in 12 cases.

### DISCUSSION

Only a few years after its initial description (Kallioniemi et al, 1992), CGH has been established as a powerful screening method in tumour genetics. Its main advantage is the detection of multiple alterations in the form of a genetic pattern within a single experiment. CGH has been used previously for the detection of aberration in SCLC (Levin et al, 1994; Ried et al, 1994). In our study, we put particular emphasis on the characterization of the deletions.

**Figure 4** Example of DNA loss on chromosome 10q assessed by LOH analysis. Cases 2 and 8 were analysed for the marker D10S1213 and case 5 for the marker D10S169. The tumour sample (T) showed only one band compared with the paired normal sample (N) of the same patient as evidence for allelic loss

### Deletions

Deletions on chromosome 3p were observed in all tumours. In the majority of them, the entire chromosomal arm was lost. Partial deletions in three cases were confined to two distinct non-overlapping regions at 3p11–14 and 3p21–pter. They co-localize with regions having previously been implicated as loci of potential tumour-suppressor genes (Hibi et al, 1992). The von Hippel–Lindau (VHL) gene at 3p25–26 is only rarely mutated in lung cancer cell lines (Sekido et al, 1994). Recently, the FHIT gene has been identified as a potential tumour-suppressor gene (Ohta et al, 1996). It is located at 3p14.2 and showed exonic losses in 80% of small-cell lung carcinomas (Sozzi et al, 1996), which makes it a prime candidate for lung carcinogenesis.

DNA loss of chromosome 10q was the second most frequent finding. This change has been observed previously (Ried et al, 1994; Levin et al, 1994), however at a lower incidence. The discrepancy compared with the other CGH studies might be explained by karyotyping errors. Chromosome 10 is difficult to identify on the basis of the chromosome banding by DAPI fluorescence. In particular, it might be confused with chromosome 8, which showed more frequent over-representations on the long arm in the previous studies. In our experience, it is helpful to employ a CGH analysis software that is based on a karyotyping program and allows the visualization of the different fluorescence signals during the karyotyping process. Particularly, the ratio image of the chromosomes representing the changes within a single metaphase facilitates the identification of homologous chromosomes, if they carry a characteristic change and, thus, reduces karyotyping errors.

We confirmed DNA loss on chromosome 10q by LOH analysis, which resulted in a similar incidence of deletions for this chromosomal arm. The minimal region of deletion was at 10q24–qter. Deletions on chromosome 10q have been described in gliomas (von Deimling et al, 1992; Leenstra et al, 1994), malignant menigeomas (Rempel et al, 1993), endometrial carcinomas (Peiffer et al, 1995) and melanoma (Isshiki et al, 1993). This change has been associated with tumour progression, which corresponds well with the fact that SCLC is generally an advanced and aggressive tumour. Recently, the MXI1 gene has been identified as a candidate tumour-suppressor gene in prostate cancer (Eagle et al, 1995). It is located at 10q24–25 and encodes a protein that negatively regulates the *myc* oncogene (Schreiber-Agus et al, 1995). This is intriguing, since amplification and overexpression of *myc* oncogenes have been reported in small-cell lung cancer (Wong et al, 1986; Brennan et al, 1991) and are associated with advanced tumour stages (Takahashi et al, 1989).

Candidate tumour-suppressor genes for the regions that are most often deleted include the retinoblastoma gene *Rb1* at 13q14 and *p53* at 17p13. In particular, DNA loss on chromosome 13q affected the locus of the *Rb1* gene at 13q14 in all except one case. This corresponds well with the fact that the Rb1 protein is frequently inactivated or lost in SCLC (Kelley et al, 1995). The incidence of DNA loss on chromosome 17p by CGH correlated with the frequency of mutations at the *p53* locus. Allelic loss and mutations of the *p53* gene occurred in 96% and 85% of tumours respectively, in a series of 27 patients with SCLC (Sameshima et al, 1992).

The role of *MCC* and *APC*, two tumour-suppressor genes that map to 5q21–22, in lung carcinogenesis has not yet been fully elucidated. Both genes have been implicated primarily in the formation of tumours in the gastrointestinal tract. Although the *APC/MCC* locus is frequently deleted in lung carcinomas, a

© Cancer Research Campaign 1997 

centromeric region at 5q13–14 (Wieland and Bohm, 1994) and a telomeric region at 5q33–35 (Hosoe et al, 1994) are additionally affected, suggesting that there are different tumour-suppressor genes on 5q associated with lung cancer.

A single paper reported 4q deletions in 6 of 16 (37.5%) SCLC cell lines (Sekido et al, 1993). Allelic loss on chromosome 4q has also been shown in hepatocellular carcinomas (Buetow et al, 1989; Urano et al, 1991) and bladder cancer, indicating two distinct regions (Polascik et al, 1995). We observed deletions in a centromeric region at 4q11–23 and a consensus telomeric region at chromosomal band 4q32. To our knowledge, no tumour-suppressor gene has yet been identified on this chromosome.

Copy number decreases occurred less frequently on chromosome 15q (55%) and chromosome 16q (45%). Deletions on chromosome 15q were recently observed by LOH analysis in multiple tumours, including carcinomas of the lung. They were associated with progression to metastatic stage in breast carcinomas (Wick et al, 1996).

### DNA over-representation

DNA amplification, being a general characteristic of tumour progression (Brison, 1993), is a frequent finding in SCLC. We observed DNA gains in particular for chromosomes 3q and 5p. In each case, they were accompanied by a DNA loss on the other chromosomal arm. The DNA gain of an entire chromosome arm may correlate to a low-level overexpression of multiple genes that reside on this chromosome arm. However, the fact that high-copy amplifications were observed at 5p11 and 3q26.3–qter probably indicates that the activation of specific proto-oncogenes contributes to a growth advantage for cells that carry a 3q or 5p gain. DNA over-representation of 3q or amplification of the telomeric part of chromosome 3q have recently been described in head and neck squamous cell carcinomas (Speicher et al, 1995; Brzoska et al, 1995).

The single most frequent high-copy amplification was mapped to chromosome 19q13, which has been previously described (Ried et al, 1994). The *fos*-B proto-oncogene is located at 19q13.3 and constitutes a candidate gene, since it is expressed in 60% of non-small-cell lung carcinomas (Wodrich and Volm, 1993). Additionally, we observed amplifications on the short arm of chromosome 19 twice. A proto-oncogene in this region is the *rel* gene that was identified by the transfection of DNA from a human melanoma cell line into NIH3T3 mouse fibroblasts. It was mapped to 19cen–p13.2 (Nimmo et al, 1991) and was overexpressed in a small proportion of human gliomas (Watkins et al, 1994).

### CGH superkaryograms in tumour classification

The sum-karyograms of the 22 SCLCs were compressed to a CGH superkaryogram (Figure 3). In this representation, the prevalent findings of the tumour class are still visible. This is promising, since it seems feasible to characterize a tumour entity or subgroup by its pattern of genetic changes, as revealed by CGH. The computer-assisted comparison of the CGH pattern of a single tumour with typical alterations of different tumour entities might become a useful adjunct in tumour classification.

The correlation between the known genetic aberrations, e.g. *Rb1* and *p53*, and the chromosomal abnormalities revealed by CGH is striking. Thus, by careful examination of the CGH patterns, it is possible to correlate certain changes with a tumour phenotype and the biological behaviour, although the causative gene defect has not yet been identified.

### Multiple changes within one chromosome

We frequently observed DNA gains and losses on the same chromosome, e.g. chromosomes 2q, 3 and 8p in Figure 1A. Such a pattern has recently been observed for chromosome 12 in gliomas by molecular genetic methods (Reifenberger et al, 1995). It is consistent with a proposed model for DNA amplification by chromosome breakage (Windle et al, 1991), and CGH is an ideal tool for the detection of this type of chromosomal instability.

Amplifications might complicate the interpretation of the allelic state, since the assessment of LOH is based on the comparison of the band intensities of the two alleles. The increased intensity of one band compared with the other might actually correspond to the amplification of one allele rather than an allelic loss. The correlations between the LOH and CGH analysis in our study are in agreement with those reported in the literature (Kallioniemi et al, 1994). Additional events that might lead to contradictory results between CGH and LOH analysis are submicroscopical deletions beyond the resolution of CGH, mitotic recombination and chromosomal loss followed by reduplication. These mechanisms have been discussed previously (Ried et al, 1994).

### Multiple changes per tumour

The study showed that deletions in general were more prevalent than amplifications. In particular, DNA under-representations of the frequently deleted loci on chromosomes 3p, 10q, 5q, 4q, 13q and 17p were each more prevalent than any of the observed over-representations. These loci were simultaneously affected in 12 cases. In our view, this is an indication that SCLC is actually defined by this pattern of deletions. The recent findings that the number of deletions correlates with the clinical outcome of breast cancer (Isola et al, 1995), renal carcinomas (Moch et al, 1996) and head and neck squamous cell carcinoma (Li et al, 1994) support the hypothesis that, in particular, the presence of multiple deletions determines the poor prognosis for SCLC patients.

### ABBREVIATIONS

CCD, charge-coupled device; CGH, comparative genomic hybridization; FITC, fluorescein isothiocyanate; TRITC, tetrarhodamine isothiocyanate; DAPI, 4,6-diamidino-2-phenylindole dihydrochloride; LOH, loss of heterozygosity; RFLP, restriction fragment length polymorphism; SCLC, small-cell lung carcinoma; SSC, saline sodium citrate buffer.

### ACKNOWLEDGEMENTS

This work was supported by the German Research Foundation (DFG) and the Berlin Cancer Society. We thank Reinhold Schäfer for his comments on the manuscript.

### REFERENCES

Brennan J, O'Conner T, Makuch RW, Simmons AM, Russell E, Linnoila RI, Gazdar AF, Ihde DC and Johnson BE (1991) *myc* family DNA amplification in 107 tumours and tumour cell lines from patients with small cell lung cancer treated with different combination of chemotherapy regimens. *Cancer Res* **51**: 1708–1721

Brison O (1993) Gene amplification and tumour progression. *Biochim Biophys Acta* **1155**: 25–41

Brzoska PM, Levin NA, FU KK, Kaplan MJ, Singer MI, Gray JW and Christman MF (1995) Frequent novel DNA copy number increase in squamous cell head and neck tumours. *Cancer Res* **55**: 3055–3059

© Cancer Research Campaign 1997

Buetow KH, Murray JC, Israel JL, London WT, Smith M, Kew M, Blanquet V, Brechot C, Redeker A and Govindarajah S (1989) Loss of heterozygosity suggests tumour suppressor gene responsible for primary hepatocellular carcinoma. *Proc Natl Acad Sci USA* **86**: 8852–8856

Cottrell S and Bodmer WF (1992) Two *MspI* polymorphisms within the *APC* gene. *Hum Mol Genet* **1**: 352

Eagle LR, Yin X, Brothman AR, Williams BJ, Atkin NB and Prochownik EV (1995) Mutation of the MXI1 gene in prostate cancer. *Nature Genet* **9**: 249–255

EL Torky M, EL Zeky F and Hall JC (1990) Significant changes in the distribution of histologic types of lung cancer. *Cancer* **65**: 2361–2367

Field JK, Spandidos DA, Steel PM, Vaughan ED, Evan GI and Moore JP (1989) Elevated expression of the *c-myc* oncoprotein correlates with poor prognosis in head and neck squamous cell carcinoma. *Oncogene* **4**: 1463–1468

Fox W and Scadding JG (1973) Medical Research Council comparative trial of surgery and radiotherapy for primary treatment of small-celled or oat-celled carcinoma of the bronchus: ten-year follow-up. *Lancet* **2**: 63–65

Ganly PS and Rabbits PH (1992*a*) Polymerase chain reaction (PCR) for detection of *MspI* polymorphism at the D3S30 locus. *Nucleic Acids Res* **19**: 3757

Ganly PS and Rabbits PH (1992*b*) Polymerase chain reaction (PCR) for detection of *BamHI* polymorphism at the THRB gene. *Nucleic Acids Res* **19**: 3757

Hibi K, Takahashi T, Yamakawa K, Ueda R, Sekido Y, Ariyoshi Y, Suyama M, Takagi H, Nakamura Y and Takahashi T (1992) Three distinct regions involved in 3p deletion in human lung cancer. *Oncogene* **7**: 445–449

Hosoe S, Ueno K, Shigedo Y, Tachibana I, Osaki T, Kumagai T, Tanio Y, Kawase I, Nakamura Y and Kishimoto T (1994) A frequent deletion of chromosome 5q21 in advanced small cell and non-small cell carcinoma of the lung. *Cancer Res* **54**: 1787–1790

Isola JJ, Kallioniemi OP, Chu LW, Fuqua Saw, Hilsenbeck SG, Osborne CK and Waldman FM (1995) Genetic aberrations detected by comparative genomic hybridization predict outcome in node-negative breast cancer. *Am J Pathol* **147**: 905–911

Isshiki K, Elder DE, Guerry D and Linnenbach AJ (1993) Chromosome 10 allelic loss in malignant melanoma. *Genes Chrom Cancer* **8**: 178–184

Johnson RE, Brerton HD and Kent CH (1978) 'Total' therapy for small cell carcinoma of the lung. *Ann Thorac Surg* **25**: 509–515

Kallioniemi OP, Kallioniemi A, Sudar D, Rutovitz D, Gray JW, Waldman F and Pinkel D (1992) Comparative genomic hybridization for molecular cytogenetic analysis of solid tumours. *Science* **258**: 818–821

Kallioniemi OP, Kallioniemi A, Piper J, Isola J, Waldman FM, Gray JW and Pinkel D (1994) Optimizing comparative genomic hybridization for analysis of DNA sequence copy number changes in solid tumours. *Genes Chrom Cancer* **10**: 231–243

Kelley MJ, Nakagawa K, Steinberg SM, Mulshine JL, Kamb A and Johnson BE (1995) Differential inactivation of CDKN2 and Rb protein in non-small-cell and small-cell lung cancer cell lines. *J Natl Cancer Inst* **87**: 756–761

Koorey DJ, Mccaughan GW, Trent RJ and Gallagher ND (1992) Dinucleotide repeat polymorphism at the D5S134 locus linked to the adenomatous polyposis coli (*APC*) gene. *Hum Mol Genet* **1**: 655

Leenstra S, Bijlsma EK, Troost D, Oosting J, Westerveld A, Bosch DA and Hulsebos TJ (1994) Allele loss on chromosomes 10 and 17p and epidermal growth factor receptor gene amplification in human malignant astrocytoma related to prognosis. *Br J Cancer* **70**: 684–689

Levin NA, Brzoska P, Gupta N, Minna JD, Gray JW and Christman MF (1994) Identification of frequent novel genetic alterations in small cell lung carcinoma. *Cancer Res* **54**: 5086–5091

Li X, Lee NK, Ye YW, Waber PG, Schweitzer C, Cheng QC and Nisen PD (1994) Allelic loss at chromosomes 3p, 8p, 13p, and 17p associated with poor prognosis in head and neck cancer. *J Natl Cancer Inst* **86**: 1524–1529

Medical Research Council Lung Cancer Working Party (1979) Radiotherapy alone or with chemotherapy in the treatment of small-cell carcinoma of the lung. *Br J Cancer* **40**: 1–10

Miura I, Graziano SL, Cheng JQ, Doyle A and Testa JR (1992) Chromosome alterations in human small cell lung cancer: frequent involvement of 5q. *Cancer Res* **52**: 1322–1328

Miyoshi Y, Nagase H, Ando H, Horii A, Shigetoshi I, Nakatsuru S, Aoki T, Miki Y, Mori T and Nakamura Y (1992) Somatic mutations of the *APC* gene in colorectal tumours: mutation cluster region in the *APC* gene. *Hum Mol Genet* **1**: 229–233

Moch H, Presti JC, Sauter G, Buchholz N, Jordan P, Mihatsch MJ and Waldman FM (1996) Genetic aberrations detected by comparative genomic hybridization are associated with clinical outcome in renal cell carcinoma. *Cancer Res* **56**: 27–30

Mori N, Yokota J, Oshima M, Cavanee WK, Mizoguchi H, Noguchi M, Shimosato Y, Sugimura T and Terada M (1989) Concordant deletions of chromosome 3p and loss of heterozygosity for chromosomes 13 and 17 in small cell lung carcinoma. *Cancer Res* **49**: 5130–5135

Nimmo ER, Sanders PG, Padua RA, Hughes D, Williamson R and Johnson KJ (1991) The *MEL* gene: A new member of the *RAB/YPT* class of *RAS*-related genes. *Oncogene* **6**: 1347–1351

Ohta M, Inoue H, Cotticelli MG, Kastury K, Baffa R, Palazzo J, Siprashvili Z, Mori M, McCue P, Druck T, Croce CM and Huebner K (1996) The FHIT gene, spanning the chromosome 3p14.2 fragile site and renal carcinoma-associated t(3;8) breakpoint, is abnormal in digestive tract cancers. *Cell* **84**: 587–597

Peiffer SL, Herzog TJ, Tribune DJ, Mutch DG, Gersell DJ and Goodfellow PJ (1995) Allelic loss of sequences from the long arm of chromosome 10 and replication errors in endometrial cancers. *Cancer Res* **55**: 1922–1926

Petersen I, Reichel M, Vogt P and Dietel M (1993) PCR-based RFLP-analysis for the detection of loss of heterozygosity on chromosomes 3p and 5q in human lung carcinomas. *J Cancer Res Clin Oncol* **119**: 60

Petersen I, Reichel MB and Dietel M (1996) Use of non-radioactive detection in SSCP, direct DNA sequencing and LOH analysis. *J Clin Pathol (Mol Pathol)* **49**: M118–M121

Polascik TJ, Cairns P, Chang WY, Schoenberg MP and Sidransky D (1995) Distinct regions of allelic loss on chromosome 4 in human primary bladder carcinoma. *Cancer Res* **55**: 5396–5399

Reifenberger G, Reifenberger J, Ichimura K and Collins VP (1995) Amplification at 12q13–14 in human malignant gliomas is frequently accompanied by loss of heterozygosity at loci proximal and distal to the amplification site. *Cancer Res* **55**: 731–734

Rempel SA, Schwechheimer K, Davis RL, Cavanee WK and Rosenblum ML (1993) Loss of heterozygosity for loci on chromosome 10 is associated with morphologically malignant meningioma progression. *Cancer Res* **53**: 2386–2392

Ried T, Baldini A, Rand TC and Ward TC (1992) Simultaneous visualization of seven different DNA probes by *in situ* hybridization using combinatorial fluorescence and digital imaging microscopy. *Proc Natl Acad Sci USA* **89**: 1388–1392

Ried T, Petersen I, Holtgreve-Grez H, Speicher MR, Schröck E, DU Manoir S and Cremer T (1994) Mapping of multiple DNA gains and losses in primary small cell lung carcinomas by comparative genomic hybridization. *Cancer Res* **54**: 1801–1806

Ries Lag, Hankey BF and Miller BA (1991) *Cancer Statistics Review, 1973–1988.* NIH publication 91–2789. National Cancer Institute: Bethesda, MD, USA.

Rodriguez E, Sreekantaiah C and Chaganti RSK (1994) Genetic changes in epithelial solid neoplasia. *Cancer Res* **54**: 3398–3406

Roth K, Hufnagl P and Wolf G (1992) AMBA/D – a new programming environment for image processing. *SPIE* **1659**: 254–261

Roth K, Wolf G, Dietel M and Petersen I (1996) Image analysis for comparative genomic hybridization (CGH) by a Windows-based karyotyping program. *Anal Quant Cytol Histol.* (manuscript submitted).

Sakurai A, Bell GI and Degroot LJ (1992) Dinucleotide repeat polymorphism in the human thyroid hormone receptor β gene (THRB) on chromosome 3. *Nucleic Acids Res* **19**: 6661

Sameshima Y, Matsuno Y, Hirohashi S, Shimosato Y, Mizoguchi H, Sugimura T, Terada M and Yokota J (1992) Alterations of the p53 gene are common and critical events for the maintenance of malignant phenotypes in small-cell lung carcinoma. *Oncogene* **7**: 451–457

Sato S, Nakamura Y and Tsuchiya E (1994) Difference of allelotype between squamous cell carcinoma and adenocarcinoma of the lung. *Cancer Res* **54**: 5652–5655

Schreiber-Agus N, Chin L, Chen K, Torres R, Rao G, Guida P, Skoultchi AI and Depinho RA (1995) An amino-terminal domain of the Mxi1 mediates anti-*myc* oncogenic activity and interacts with a homolog of the yeast transcriptional repressor SIN3. *Cell* **80**: 777–786

Sekido Y, Takahashi T, Ueda R, Takahashi M, Suzuki H, Nishida K, Tsukamoto T, Hida T, Shimokata K, Zsebo KM and Takahashi T (1993) Recombinant human stem cell factor mediates chemotaxis of small-cell lung cancer cell lines aberrantly expressing the c-kit protooncogene. *Cancer Res* **53**: 1709–1714

Sekido Y, Bader S, Latif F Gnarra JR, Gazdar AF, Linehan WM, Zbar B, Lerman MI and Minna JD (1994) Molecular analysis of the von Hippel–Lindau disease tumour suppressor gene in human lung cancer cell lines. *Oncogene* **9**: 1599–1604

Sozzi G, Veronese ML, Negrini M, Baffa R, Cotticelli MG, Inoue H, Tornielli S, Pilotti S, De Gregorio L, Pastorino U, Pierotti MA, Ohta M, Huebner K and Croce CM (1996) The FIHT gene at 3p14.2 is abnormal in lung cancer. *Cell* **85**: 17–26

© Cancer Research Campaign 1997

Speicher MR, Howe C, Crotty P, Du Manoir S, Costa J and Ward DC (1995) Comparative genomic hybridization detects novel deletions and amplifications in head and neck squamous cell carcinomas. *Cancer Res* **55**: 1010–1013

Takahashi T, Obata Y, Sekido Y, Hida T, Ueda R, Watanabe H, Ariyoshi Y, Sugiura T and Takahashi T (1989) Expression and amplification of *myc* gene family in small cell lung cancer and its relation to biological characteristics. *Cancer Res* **49**: 2683–2688

Tsuchiya E, Nakamura Y, Weng S-Y, Nakagawa K, Tsuchiya S, Sugano H and Kitagawa T (1992) Allelotype of non-small cell lung carcinoma – comparison between loss of heterozygosity in squamous cell carcinoma and adenocarcinoma. *Cancer Res* **52**: 2478–2481

Urano Y, Watanabe K, Lin CC, Hino O and Tamaoki T (1991) Interstitial chromosomal deletion within 4q11–q13 in a human hepatoma cell line. *Cancer Res* **51**: 1460–1464

Von Deimling A, Louis DN, Von Ammon K, Petersen I, Höll T, Chung RY, Martuza RL, Schoenfeld DA, Yasargil MG, Wiestler OD and Seizinger BR (1992) Association of epidermal growth factor receptor gene amplification with loss of chromosome 10 in human glioblastoma multiforme. *J Neurosurg* **77**: 295–301

Watkins D, Dion F, Poisson M, Delattre JY and Rouleau GA (1994) Analysis of oncogene expression in primary human gliomas: evidence for increased expression of the *ros* oncogene. *Cancer Genet Cytogenet* **72**: 130–136

Whang-Peng J, Kao-Shan CS, Lee EC, Bunn PA, Carney DN, Gazdar AF and Minna JD (1982) Specific chromosome defect associated with human small-cell lung cancer: deletion 3p(14–23). *Science* **215**: 181–182

Wick W, Petersen I, Schnitzler R, Wolfarth B, Lenartz D, Bierhoff E, Hümmerich J, Stangl AP, Schramm J, Wiestler OD and Von Deimling A (1996) Evidence for a novel tumour suppressor gene on chromosome 15 associated with progression to metastatic stage in breast cancer. *Oncogene* **12**: 819–823

Wieland I and Bohm M (1994) Frequent allelic deletion at a novel locus on chromosome 5 in human lung cancer. *Cancer Res* **54**: 1772–1774

Windle BE, Draper BW, Yin Y, O'Gorman S and Wahl GM (1991) A central role for chromosome breakage in gene amplification, deletion formation, and amplicon integration. *Genes Dev* **5**: 160–174

Wodrich W and Volm M (1993) Overexpression of oncoproteins in non-small cell lung carcinomas of smokers. *Carcinogenesis* **14**: 1121–1124

Wolf G, Petersen I, Roth K and Dietel M (1995) Computergestütztes Programm für die molekulare zytogenetische Analyse von soliden Tumoren mittels vergleichender genomischer Hybridisierung (CGH) und digitaler Bildverarbeitung. *Verh Dtsch Ges Path* **79**: 1807

Wong AJ, Ruppert JM, Eggleston J, Hamilton SR, Baylin SB and Vogelstein B (1986) Gene amplification of *c-myc* and *N-myc* in small cell carcinoma of the lung. *Science* **233**: 461–464

Yokota J, Wada M, Shimosato Y, Terada M and Sugimura T (1987) Loss of heterozygosity on chromosomes 3, 13 and 17 in small cell carcinoma and on chromosome 3 in adenocarcinoma of the lung. *Proc Natl Acad Sci USA* **84**: 9252–9256

Yokota J, Tsukada Y, Nakajima T, Gotoh M, Shimosato Y, Mori N, Tsunokawa Y, Sugimura T and Terada M (1989) Loss of heterozygosity on the short arm of chromosome 3 in carcinoma of the uterine cervix. *Cancer Res* **49**: 3598–3601

© Cancer Research Campaign 1997